# Effect of output variation with dose rate on the Virtual Wedge factor

**DOI:** 10.1120/jacmp.v9i1.2784

**Published:** 2008-01-28

**Authors:** X. Ronald Zhu, Michael T. Gillin

**Affiliations:** ^1^ Department of Radiation Physics The University of Texas M.D. Anderson Cancer Center Houston Texas U.S.A.

**Keywords:** Virtual Wedge, quality assurance

## Abstract

The Siemens Virtual Wedge factor (VWF: Siemens Medical Solutions, Malvern, PA) may drift significantly because of an increase in output as the dose rate declines. This variation in output is caused by sample and hold boards in the dosimetry circuit that become defective because of radiation damage. Here, we present a simple model based on the principle of Virtual Wedge operation and the measured output variation with dose rate to quantitatively describe VWF drift as a function of field size and wedge angle. Our results support the recommendation that VWF be measured for large field sizes (for example, 20×20 cm) and large wedge angles (for example, 60 degrees) as a part of routine quality assurance.

PACS numbers: 87.55.Qr, 87.55.N‐

## I. INTRODUCTION

Siemens Virtual Wedge (VW: Siemens Medical Solutions, Malvern, PA) creates a wedge‐shaped dose distribution by moving one collimator jaw across the field during irradiation.^(1)^ For a given VW field, the speed of the jaw is constant, but the dose rate varies as the moving jaw travels through the field.

Recently, Faddegon and Garde^(2)^ reported a significant drift in the VW factor (VWF) for large fields delivered with a 60‐degree wedge for 5 of 6 linear accelerators in their clinic. The drift was traced to variation in the pulse rate (dose rate) caused by defective sample and hold boards in the dosimetry circuit. The report discussed the effect of VWF drift on the quality assurance (QA) program; however, the relationship between output variation with dose rate and VWF was not quantified. In spring 2007, Malkoske and Nakonechny^(3)^ presented a detailed analysis of the influence of monitor chamber calibration on VW dosimetry. We also observed VWF drift, especially for large wedge angles and field sizes. Here, we present a simple model, based on the principle of VW operation, to quantitatively describe VWF drift as a function of field size and wedge angle.

## II. METHODS

An exponential function can be used to analytically describe the cumulative monitor units (MUs) in the VW direction.^(1)^ The delivery of a VW field can be described as follows:

- Just before beam delivery begins, the moving jaw moves toward the fixed jaw with a gap of width $l_{\text{gap}}$ (typically 1 cm) between the two jaws.
- With the moving jaw parked at this position, a few MUs are delivered at the maximum dose rate available for the beam, ${\left(d\text{MU}/dt\right)}_{mach,\max}$.
- The moving jaw then travels across the field at a constant speed, and the dose rate varies according to the formula
  (1) $$\frac{dMU(y)}{dt}={\left(\frac{dMU}{dt}\right)}_{march,\text{max}}\text{exp}[-c\mu \;\text{tan}(\alpha )(y-y_{\text{min}}-l_{gap})],$$
  where *y* is the position of the moving jaw (y=0 is the central axis), $y_{\min}$ corresponds to the fixed jaw position, μ is the default mean linear attenuation coefficient, *c* is the calibration factor for μ, and α is the nominal wedge angle. When the moving jaw reaches the maximum opening, $y_{\max}$, the dose rate becomes the lowest for the field.
- As soon as the moving jaw arrives at $y_{\max}$, the machine switches back to the maximum dose rate to deliver the remaining MUs.

When the output varies with the dose rate, equation 1 can be used to relate this variation to the moving jaw's position, yielding

(2) $$OP(MU/\text{min})=OP\left(\frac{dMU(y)}{dt}\right)=OP(y),$$

where OP is the output. The total change of output as measured along the central axis, y=0, because of the variation of output with dose rate can be written

(3) $$\Delta OP(\alpha ,y_{\text{min}},y_{\text{max}})=\int_{0}^{Y_{\text{max}}}[OP_{d}(y)-OP_{n}(y)]\frac{dMU(y)}{dy}dy,$$

where ${\text{OP}}_{d}$ and ${\text{OP}}_{n}$ are output factors for the defective and normal dosimetry circuit boards, respectively, and

(4) $$\frac{dMU(y)}{dy}=MU(0)(-c\mu \;\text{tan}\;\alpha )\text{exp}[-c\mu \;\text{tan}(\alpha )y],$$

is the MU differential delivered at the *y* position. Then, the change of VWF attributable to variation of dose rate can be expressed as

(5) $$\Delta VWF(\alpha ,y_{\text{min}},y_{\text{max}})=\frac{\Delta OP(\alpha ,y_{\text{min}},y_{\text{max}})}{OP_{open}(y_{\text{min}},y_{\text{max}})},$$

where ${\text{OP}}_{\text{open}}$ is the output for the corresponding open field. Equation 5 has no fitting parameter; the needed data are the measured output as a function of dose rate for the defective and normal circuit boards, and the parameters for VW operation (the maximum dose rate of the beam, the attenuation coefficient μ, calibration factor *c*, field size, and wedge angle).

A 6‐MV beam from a Siemens MD2 linear accelerator with the VW option was used in the present work. For that beam, $\mu =0.0507\;{\text{cm}}^{-1}\;\text{and}\;c=1.10$. For the symmetric field sizes used here, $l_{\text{gap}}=1$ cm. For a 20×20 cm square field, for example, $y_{\min}=-10\;\text{cm}\;\text{and}\;y_{\max}=10\;\text{cm}$. This machine provides only one VW dose rate range: 30 – 200 MU/min. The VWF was defined as the ratio of the dose measured on the central axis for the wedge field to that for the open field. Wedge factors were measured at a depth of 10 cm using a Farmer‐type ion chamber placed in a sealed water phantom with its axis parallel to the non‐wedge direction. The target‐to‐phantom surface distance was 100 cm. The measured VWF was an average of two wedge orientations, one with Y1 as the moving jaw (1VW), and the other with Y2 as the moving jaw (2VW). The relative output as a function of dose rate was measured in the same geometry using a field size of 10×10 cm for sets of normal and defective sample and hold boards for the dosimetry circuit. The variable dose rates were achieved by adjusting the pulse repetition frequency programmed on the Siemens accelerator; the beam was delivered in the “open loop” mode with all relevant dosimetry interlocks overridden.

The results of output variation as a function of dose rate were fitted to fourth‐order polynomials so that equation 2 could be used to relate them to the VW variable dose rate and moving jaw position. Equation 3 was numerically integrated using a simple trapezoid method.

## III. RESULTS AND DISCUSSION

**Figure 1:**
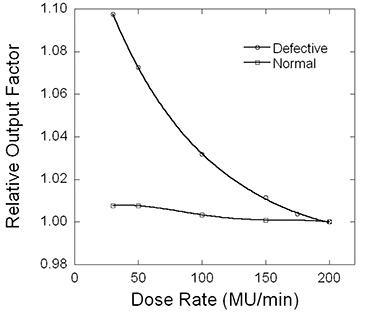
Relative output of the 6‐MV beam as a function of dose rate for sets of normal and defective sample and hold boards for the dosimetry circuit. The solid lines are fourth‐order polynomial fits of the measured data. MU=monitor unit.

Fig. 1 shows measured output as a function of dose rate (in monitor units per minute) for sets of normal and defective sample and hold dosimetry circuit boards, normalized to the dose rate of 200 MUs/min. Because the dose rate varied from 200 MUs/min to 30 MUs/min, the change of output was significantly different between the normal and defective circuit boards (1% vs. 10%). Table 1 lists the difference in 60‐degree VWF as a function of field size, and Table 2 lists the difference in VWF for a field size of 20×20 cm as a function of wedge angle between the normal and defective circuit boards. Tables 1 and 2 also show the model results predicted by equation 5 based on the data in Fig. 1.

**Table 1:** Measured and calculated change in the Siemens Virtual Wedge factor (VWF: Siemens Medical Solutions, Malvern, PA) for a 60‐degree Virtual Wedge as a function of field size

	Change of VWF	
Field size (cm)	Measured	Calculated
5	−0.001	0.002
10	0.008	0.009
15	0.021	0.022
20	0.036	0.037

**Table 2:** Measured and calculated change in the Siemens Virtual Wedge factor (VWF: Siemens Medical Solutions, Malvern, PA) for a 20×20 cm field as a function of wedge angle

	Change of VWF	
Wedge angle (deg.)	Measured	Calculated
15	0.001	0.001
30	0.003	0.005
40	0.009	0.010
45	0.014	0.014
50	0.019	0.019
55	0.025	0.026
60	0.036	0.037

The simple model developed here predicted the change in VWF very well regardless of whether it was analyzed in terms of field size or wedge angle. As dose rate varied from 200 MUs/min to 30 MUs/min, a 10% increase in output resulted in an approximately 4% increase in VWF for the 20×20 cm field delivered by the 6‐MV beam with a 60‐degree wedge. For the 60‐degree VW, the change in VWF was less than 2% for field sizes less than 15×15 cm. Given a field size of 20×20 cm, the change in VWF was greater than 2% only if the wedge angle was larger than 50 degrees.

The relationship between field size and wedge angle can easily be understood by using equation 1 for the 6‐MV beam delivering 100 MU on the MD2 linear accelerator as follows:

- For a 60‐degree VW, the slowest dose rates are 135.9 MUs/min and 31.9 MUs/min for, respectively, field sizes of 5 cm^2^ and 20 cm^2^ in the moving jaw direction.
- For a field size of 20 cm^2^ in the moving jaw direction, the slowest dose rates are 115.8 MUs/min and 31.9 MUs/min for, respectively, VW angles of 15 degrees and 60 degrees.

When using large field sizes and wedge angles, the dose rate of VW delivery can be very small. The increased output at low dose rates (as shown in Fig. 1) contributes more to VWF drift. Therefore, large field sizes and large wedge angles are more sensitive in detecting the VWF drift. It should be pointed out that, for small wedge angles and low MUs, the dose rate may also be low, but the fraction of dose delivered with the low dose rate in the sweeping phase relative to the open field is small. Therefore, the effect is not as profound as for large wedge angles and large field fields.

## IV. CONCLUSIONS

We developed a simple model to predict the drift in VWF as a function of field size and wedge angle. This model provides a quantitative explanation of VWF drift caused by the variations in output with dose rate, which may be caused by radiation damage to the sample and hold boards in the dosimetry circuit. Faddegon and Garde^(2)^ empirically related VWF drift to output variation at two dose rate points (the maximum dose rate and reduction by a factor of 5 in the maximum dose rate). We demonstrated that it is possible to predict the drift in VWF if output as a function of dose rate for normal and defective circuit boards is known. We are not suggesting that the method reported here be used as a routine QA procedure; rather, we are hoping that it provides a different angle from which to look at the problem of VWF drift. Our results support the recommendation^(2)^ that VWF be measured for large field sizes (for example, 20×20 cm) and large wedge angles (for example, 60 degrees) as a part of routine QA.

It should be pointed out that the defective circuit boards in this work were used only for the purpose of demonstrating the problem of VWF drift. We are not by any means suggesting the clinical use of defective boards. The QA program should detect this type problem, and the defective boards should be replaced promptly. Regular QA of VWF for large field sizes and large wedge angles should provide an additional way to identify this problems of circuit boards.

## ACKNOWLEDGMENTS

The experimental data were measured while the authors were working at the Medical College of Wisconsin in Milwaukee. Editorial review by Kathryn Carnes of the M.D. Anderson Department of Scientific Publications is greatly appreciated.
